# Morphed and moving: TNFα-driven motility promotes cell dissemination through MAP4K4-induced cytoskeleton remodeling

**DOI:** 10.15698/mic2014.05.146

**Published:** 2014-04-24

**Authors:** Min Ma, Martin Baumgartner

**Affiliations:** 1 Neuro-Oncology Laboratory, Experimental Infectious Diseases and Cancer Research, University Children’s Hospital Zürich, 8008 Zürich, Switzerland.; 2 Graduate School for Cellular and Biomedical Sciences, University of Bern, Switzerland.

**Keywords:** Theileria annulata, motility, dissemination, invasiveness, MAP4K4, TNFα, ERM proteins, actin remodeling

## Abstract

Cell dissemination from an initial site of growth is a highly coordinated and controlled process that depends on cell motility. The mechanistic principles that orchestrate cell motility, namely cell shape control, traction and force generation, are highly conserved between cells of different origins. Correspondingly, the molecular mechanisms that regulate these critical aspects of migrating cells are likely functionally conserved too. Thus, cell motility deregulation of unrelated pathogenesis could be caused and maintained by similar mechanistic principles. One such motility deregulation disorder is the leukoproliferative cattle disease Tropical Theileriosis, which is caused by the intracellular, protozoan parasite *Theileria annulata*. *T. annulata* transforms its host cell and promotes the dissemination of parasite-infected cells throughout the body of the host. An analogous condition with a fundamentally different pathogenesis is metastatic cancer, where oncogenically transformed cells disseminate from the primary tumor to form distant metastases. Common to both diseases is the dissemination of motile cells from the original site. However, unlike metastatic cancer, host cell transformation by *Theileria* parasites can be reverted by drug treatment and cell signaling be analyzed under transformed and non-transformed conditions. We have used this reversible transformation model and investigated parasite control of host cell motile properties in the context of inflammatory signaling in Ma M. *et al.* [PLoS Pathog (2014) 10: e1004003]. We found that parasite infection promotes the production of the inflammatory cytokine TNFα in the host macrophage. We demonstrated that increased TNFα triggers motile and invasive properties by enhancing actin cytoskeleton remodeling and cell motility through the ser/thr kinase MAP4K4. We concluded that inflammatory conditions resulting in increased TNFα could facilitate cell dissemination by activating the actin cytoskeleton regulatory kinase MAP4K4. We discuss here the relevance of TNFα-MAP4K4 signaling for pathogen-driven cell dissemination and its potential impact on the induction of metastasis in human cancer.

## PARASITE-ENFORCED ACQUISITION OF MOTILE PROPERTIES AND ITS ANALYSIS

The propagation of parasites inside their host or from one host to the next requires the acquisition of motile properties. In the case of intracellular parasitism, these properties can be triggered in the host cell, which allows the parasite to spread stealthily and protected from the immune system. This parasite-induced host cell dissemination and pathogen dispersion was referred to as Trojan horse strategy. Unlike the mythological horse, however, which had to be dragged into the city of Troy, parasitized host cells move autonomously. This is particularly striking in the case of dendritic cells, which within minutes of *Toxoplasma* or *Neospora* infection begin to migrate rapidly. Macrophages infected with *Theileria annulata* migrate *in vitro* and *in vivo*, whereby migration is parasite dependent because its elimination with the parasiticidal drug buparvaquone (BW720c) markedly alters the morphological and migratory properties of the host cells. Host cell mobilization by the parasite requires an exchange between the parasite and host cell signaling but our understanding of parasite molecules controlling host cell functions remained marginal due to technical obstacles preventing the genetic manipulation of the parasite. However, the parasite can be experimentally eliminated by BW720c treatment and with it the source of promigratory signaling be disabled. This allows comparing motile behavior of parasite-infected with drug-cured cells of the same genetic background and characterizing host cell mechanisms needed for infected cell mobilization. Using such a comparative approach we have characterized *T. annulata*-dependent morphological and functional alterations in the host cell and investigated the underlying signaling pathways and molecular effectors.

## AUTOCRINE TNFα MOBILIZES PARASITE-INFECTED CELLS

Progression of Tropical Theileriosis and morbidity caused by *T. annulata* depends on the susceptibility of the host to the parasite, which is due in part to parasite-induced secretion of cytokines, including GM-CSF, TGFβ or TNFα. The comparison of susceptible with resistant animals by the labs of Elizabeth Glass and Gordon Langsley revealed a susceptibility signature of cytokine expression. In parallel, it became clear that several factors secreted by infected cells must contribute to infected cell dissemination, some of which (e.g., TGFβ) markedly increased in susceptible animals in response to infection. TNFα expression on the other hand was also increased upon infection but independent of the host’s susceptibility to the disease. Consistent with the causative role of *T. annulata*, TNFα expression decreased drastically when the intracellular parasite was eliminated by drug treatment. A consequence of parasite elimination is the change in cell morphology and in the number of lamellipodia, which are filamentous-actin (F-actin)-rich structures at the leading edge of migrating cells that enable protrusion, adhesion and invasion. We hypothesized that a novel function of TNFα could be to stimulate morphodynamic processes controlling cell motility. Indeed, depletion and complementation experiments altering TNFα abundance clearly confirmed a general effect of TNFα on morphodynamic processes and cell motility. Intriguingly, by simply decreasing TNFα abundance, we could reduce invasive motility of the infected cells, which indicated for the first time the potentially critical role of TNFα in cellular control of invasiveness. We ascribed the reduced invasiveness in the absence of TNFα to impaired F-actin assembly and maintenance in protrusive cellular invasion structures. We had previously shown that the assembly, maintenance and turnover of F-actin-rich protrusive invasion structures such as lamellipodia, podosomes and membrane blebs determine the efficacy of migration of *T. annualata *infected cells in three-dimensional matrices. Antonio Barragan’s group, who revealed massive F-actin dynamics in *Toxoplasma*-infected dendritic cells, noted analogous observations under standard culture conditions. Thus, the spatio-temporal control of F-actin polymerization and turnover determines whether and how infected cells migrate and our data implicated TNFα at the origin of this process.

## MAP4K4 DIVERTS TNFα SIGNALS TOWARDS CELL MOTILITY REGULATION

How could TNFα control actin dynamics? TNFα signals through TNFα-receptor 1 and 2 to promote proliferation and survival or to activate pathways that either trigger apoptotic or necroptotic cell death. These signals are transmitted through at least three distinct pathways, one of which involving the activation of the c-jun N-terminal kinase JNK. JNK is permanently activated at low levels in *Theileria*-infected cells and the Langsley lab has shown that JNK signaling is essential for survival and metastasis of *Theileria*-infected cells. TNFα can activate JNK through the serine/threonine kinase MAP4K4 to mediate inflammatory and metabolic processes. MAP4K4, a mechanistically relatively poorly understood molecule, has in recent years emerged as a key player in inflammatory and migratory processes including cancer progression. While trying to understand how these individual evidences may be connected, we began considering MAP4K4 as a potential hub diverting TNFα signals towards effectors that control F-actin dynamics and cell motility. We experimentally tested this possibility in *T. annulata*-infected cells and found that MAP4K4 indeed mediated the motile and invasive processes induced by TNFα. Rather unexpectedly, we also found that TNFα specifically activated the F-actin-plasma membrane cross-linker proteins of the ezrin, radixin, moesin (ERM) family and more generally increased F-actin assembly in cells, whereby both processes were impaired when MAP4K4 was depleted. From these studies we concluded that the increased motility and invasiveness we observed under conditions of chronically increased TNFα are the consequence of signal bifurcation at the level of MAP4K4, which ultimately couples inflammatory signaling to the regulation of actin dynamics and cell motility.

## DOES TNFα CAUSE INVASIVE MIGRATION OF HUMAN CANCER CELLS?

**Figure 1:**
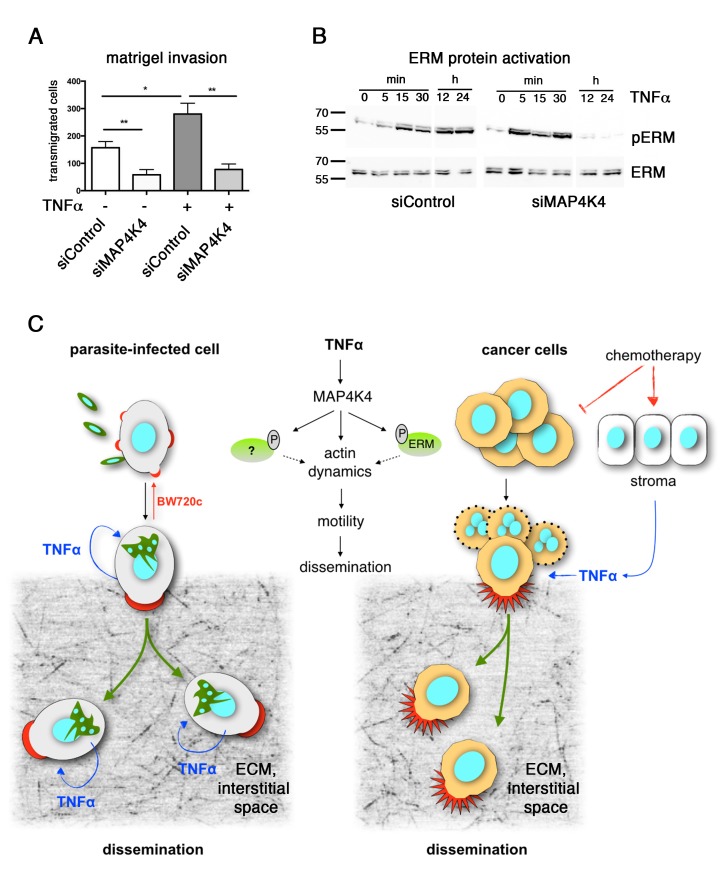
FIGURE 1: **(A)** Control and MAP4K4-depleted MDA-MB231 breast cancer cells were analyzed in Boyden chamber transwell matrigel invasion assay. TNFα stimulation (25 ng/ml) significantly increases matrigel invasiveness of MDA-MB231 cells. If the potential proto-oncogenic ser/thr kinase MAP4K4 is depleted, invasive cell motility is largely blocked both under unstimulated as well as under TNFα stimulated conditions. **(B)** The downstream effector proteins of the ERM family are activated (phosphorylated) in response to TNFα stimulation (25 ng/ml) in MDA-MB231. Depletion of MAP4K4 blunts their activation. **(C) **Schematic overview of the proposed mechanistic linkage between TNFα stimulation and invasive cell motility. ECM: extracellular matrix.

Evidently, *T. annulata*-infected and transformed macrophages are different from metastatic cancer cells in several ways. Common to both, however, is the capability to disseminate and to breach tissue and extracellular matrix barriers. Our study revealed that invasive motility is driven by the permanent exposure of the infected cells to TNFα, which triggers and maintains F-actin assembly and turnover to drive cell movement. Could inflammation, in particular TNFα, also fuel dissemination of human cancer cells? The link between chronic inflammation, such as gastritis or hepatitis and cancer, has long been established and TNFα has emerged as a suspect of promoting cancer progression under these conditions. Moreover, a recent publication by Joan Massagué’s laboratory in breast cancer research showed that chemotherapeutics trigger the release of TNFα from stromal cells and that this TNFα release helps breast cancer cells to survive and metastasize. We therefore tested the possibility that breast cancer cells respond to TNFα with migration and invasion. Interestingly, analogous to *T. annulata* infected macrophages, MDA-MB231 breast cancer cells showed significantly increased motile and invasive properties when stimulated with TNFα (Fig. 1A). Importantly, these properties were blunted when MAP4K4 was depleted. Additionally TNFα stimulation of MDA-MB231 cells promoted the C-terminal phosphorylation of ERM proteins (Fig. 1B). Again, MAP4K4 was necessary for long term activation of ERM proteins in response to TNFα, combined suggesting that TNFα activation of cytoskeleton dynamics through MAP4K4 is functionally conserved.

Clearly, more in-depth analysis will be needed to fully clarify the functional significance of TNFα-MAP4K4 signaling for cancer cell progression (Fig. 1C). However, our study of host cell exploitation by an intracellular pathogen has revealed an interesting link between inflammatory cytokine signaling and cell mobilization, which may also be relevant in cancer metastasis and immune cell mobilization under conditions of chronic inflammation such as rheumatoid arthritis.

